# A New Tool for an Awareness Plan Concerning Critical Issues, Needs and Attitudes of Citizens on the Use of Medicines

**DOI:** 10.3390/healthcare9111409

**Published:** 2021-10-20

**Authors:** Barbara Pittau, Piergiorgio Palla, Francesca Pettinau, Antonio Mastino

**Affiliations:** Institute of Translational Pharmacology, National Research Council, 00133 Rome, Italy; palla.pg@gmail.com (P.P.); f.pettinau@tiscali.it (F.P.)

**Keywords:** medicines, communication, citizens, attitudes, software application, awareness plan

## Abstract

This article describes a pilot study to test the adequacy of a newly developed tool for an awareness plan on the importance of properly using pharmaceuticals. The new tool consists of face-to-face interviews with adult citizens on their approach to the use of medicines and of the following data analysis with a dedicated software application. The pilot study was carried out in a sample area of Sardinia, in Italy. The data from the interviews collected anonymously and analysed in aggregate actually emphasised the critical issues and needs in the use of pharmaceuticals in the sample area involved, also encouraging communication among different actors. The pilot study revealed that the designed tool could represent a novel strategy to stimulate interchanges of information on the proper use of pharmaceuticals with a potential impact on people’s health.

## 1. Introduction

The importance of health information and related instruments aimed to positively influence change in people’s behaviour, such as information campaigns, intervention planning and intervention evaluation, have been considered as relevant factors for health promotion [1,2,3,4]. In particular, encouraging communication concerning the importance of the appropriate use of pharmaceuticals among the interested parties, such as citizens, pharmacists, sanitary staff or scientists, should have important implications for individual health and healthcare system’s sustainability. In fact, the importance of actions capable of increasing the awareness of the potential risks in the use of drugs, which must be balanced with respect to the benefits, has been highlighted by international and national regulatory agencies as well as by the scientific community [1,5,6,7,8,9]. Moreover, the same and other reports stressed the centrality of citizens who can provide valuable information on the safety of drugs and the relevance of collaboration between patients and healthcare professionals, also pointing out the potential positive impact on pharmacovigilance [9,10]. Indeed, despite their potential impact on public health, critical issues concerning household use of medicines have not been systematically investigated [11,12]. In this scenario, the understanding of the context has been shown to be essential for an initiative aimed to ameliorate the proper use of pharmaceuticals to get a chance to be effective [4,13,14,15,16]. The applied approaches to this issue have been generally focused on information campaign/educational intervention or on software application/informatics products to support people’s health. Our intent was to merge these aims in a single goal.

Based on the considerations reported above, an oral interview-based awareness plan directed at adult citizens (≥18 years) on the importance of using pharmaceuticals taking into consideration the risks and benefits of properly following the indications by healthcare professionals was designed. To preliminarily investigate the efficiency and the benefits of the plan and to test a specific informatics tool, the pilot study reported here was carried out. To this purpose, a territory was selected that included a large user base with different subareas and the presence of different kinds of healthcare facilities, thus potentially including different needs. Moreover, sampling was planned to detect and represent variability that could occur in larger areas. The strategy employed during the pilot study was original in terms of the software application and of the oral interview designed and used. In particular, the oral interview approach was selected in order to give time to the respondent to reflect on their own attitude and experience concerning the use of medicines, also allowing for the possibility that doubt arose.

The main aspects of the tool we wanted to test with this pilot study were as follows: (i) the capability of the developed software to manage data and present them in a summarised format capable of providing intelligible answers and (ii) the capability of the interview to impact the approach of the respondents to medicines. The data collected during the pilot study not only confirmed the efficiency of the utilised tool, but provided new information on the problems and needs inherent to the approach of citizens to drugs, also promoting communication among the interested actors.

## 2. Materials and Methods

### 2.1. Data Collection and Analysis

The pilot study for the awareness plan was based on a new tool consisting of an integrated double approach. The first arm of the approach was the design and implementation of a new relational database-backed web application named COLLABORAFARMACISOLA (i.e., the Italian for “The island collaborates on pharmaceuticals”, hereinafter referred to as COFAIS) as a major instrument to analyse, anonymously and in aggregate, the data obtained by means of oral interviews. This software application allows collecting and storing information and processing and visualising it through a set of options to support the analysis and highlight insights. The second arm of the integrated approach consisted of the oral interviews themselves. The face-to-face interview was conducted with an initial open approach, later progressively focusing on different topics (knowledge, experiences, opinions and approach of the respondent towards medicines) expressed with defined open and closed questions in a specified order of presentation. The followed logic was to ask questions, also including the possibility that doubts and needs could be written down.

### 2.2. Study Context

Each interview was always proposed to a citizen directly by a researcher of the group that developed the plan between the years 2016–2017 in various waiting rooms of different healthcare facilities: hospitals, health centres, pharmacies and offices of general practitioners. In particular, interview spaces were placed in areas open to the public and at the same time allowing a face-to-face dialogue. Local health services, local pharmacists and health professionals who found the initiative useful authorised its implementation in their areas of relevance. Logistical support for the initiative also came from some local municipalities and cultural spots. At the beginning of the interview, it would be specified that the study was carried out by a public research organisation with financial support from Fondazione di Sardegna. To address privacy regulations, it was explained that the dialog was voluntary and that the collected information had the only purpose of the research to contribute to the public benefit. The interviewer would also clarify that all the data were collected anonymously and analysed in aggregate to prevent the tracing back to particular individuals. No information about the citizens’ health conditions was considered. The interview did not interfere at all with the patient–healthcare professional relationship. Moreover, during the interview, the importance of reporting doubts and emerging problems to the pharmacist, general practitioner or the specialist doctor that suggested or prescribed the drugs was highlighted. Thus, the pilot study was organised, as a whole, with the typical features of an awareness plan.

The pilot study was carried out in an area of southwestern Sardinia (island, centre of Italy) with approximately 100,000 inhabitants, of which about 85,000 were adults. Posters and websites illustrating the plan and its aim were advertised in the sample area involved. Attention was given to sampling taking into consideration the different subareas present in the sample area as a whole.

## 3. Results

**Figure 1:**
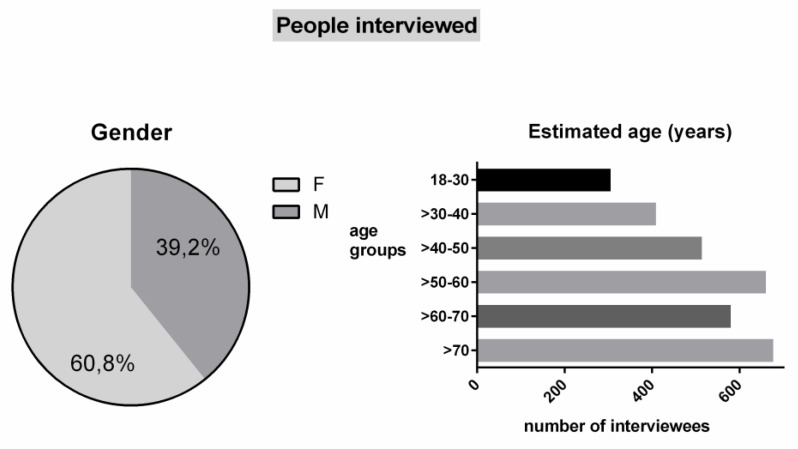
Charts of the people interviewed divided by gender and estimated age by the COFAIS platform. Sample size, *n* = 3145.

The most representative results of the pilot study are detailed in this report to give a comprehensive idea of the potential strength of the newly designed implementation plan. Note that the original language utilised by the COFAIS platform was Italian and all the Figures shown are the translations to English of the original ones generated by the platform. Figure 1 shows the chart generated by the COFAIS platform which summarises the number of people interviewed (more than three thousand interviews) distinguished by gender and age (as estimated by the interviewer). The chart represents population distribution deriving from random sampling that looks qualitatively representative as the analysis showed that the answers were gender-independent overall. The exception was found for the sampled age range > 40–50 (lower than expected according to the demographic statistics); this could be caused by bias in age estimation.

**Figure 2:**
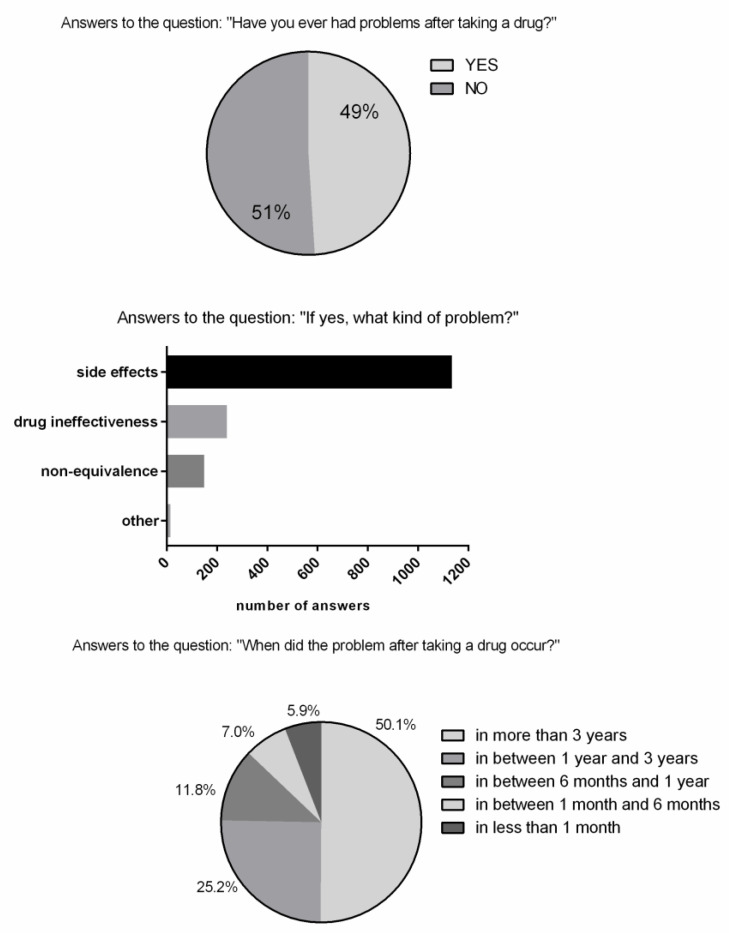
Answers related to the question “Have you ever had problems after taking a drug?” Sample size, *n* = 3145.

One of the aspects that was investigated was subjective experience towards pharmaceuticals in terms of side effects, non-equivalence, drug ineffectiveness, etc. Figure 2 shows that 49% of the interviewees answered “yes” for having had in their own subjective experience a problem after taking a drug. Most of answers referred to an experience that occurred more than one year before. About 83% of those who answered “yes” for having had a problem said that they were not taking other drugs or natural products in the same period (Supplementary Materials, Figure S1). Moreover about 86% of those who answered “yes” for having had a problem after taking a drug said that they verified the drug expiration dates (chart not shown). As indicated in the section of the COFAIS platform where the aggregated data on the drugs could be checked, the most frequently pointed out pharmaceuticals for potential problems were the following: drugs with ketoprofen as the active ingredient (side effects), drugs with nimesulide as the active ingredient, and in particular generic nimesulide drugs, or inhibitors of the gastric proton pump, in particular generic omeprazole drugs (non-equivalence after medication switching) (Supplementary Materials, Figure S2). To improve the understanding concerning the generic drugs pointed out by the respondents, additional questions were designed. Ninety percent of the ones who answered “yes” for previously taking a branded drug (about 71%) said that they did not have problems after taking those (Supplementary Materials, Figure S3).

**Figure 3:**
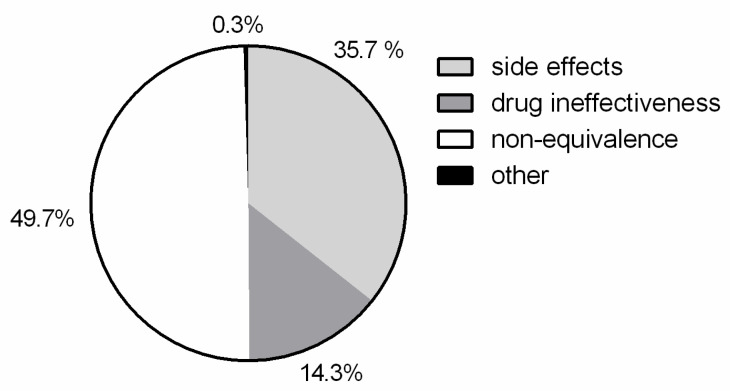
Answer chart for the analysis of potential problems concerning generic drugs. Sample size, *n* = 294.

Thanks to the possibility given by the software platform of crossing the data of the different questions/answers, it was evinced that the higher percentage of potential problems of generic drugs was related to non-equivalence (49.7%) (Figure 3). Moreover, based on the respondent answers, more than 80% of the reported cases of potential problems were not related to the concurrent intake of other drugs (chart not shown).

The respondents were also asked to report treatment interruptions, self-use without healthcare professional consultation, attention to reading the patient information leaflet and awareness of the economic value of pharmaceuticals. Based on the given answers, about 33% of the respondents took pharmaceuticals without consulting healthcare providers, 22% of them interrupted treatment by themselves and about 14% did not check before buying a drug if it was already available at home (Supplementary Materials, Figure S4). The most frequently indicated pharmaceutical products during this part of the interview were nonsteroidal anti-inflammatory drugs. The frequency of taking drugs in this way was reported to be mostly “one or more times a year”, although more than 100 respondents said “one or more times a month” (chart not shown). A focus on perception of the importance of the patient information leaflet on about 500 respondents was also performed (random sampling over a shorter period of time). About 70% of the interviewees said they did not read the information leaflet. A very low percentage of the respondents (about 2%) said that they noticed the simplification introduced a few years before concerning the information leaflet in accordance with the European and national guidelines on readability (chart not shown) [17]. The attitude towards the use of generic drug products was also investigated. About 36% of the respondents professed they did not use generic drugs (Supplementary Materials, Figure S5).

The aspects on which the respondents expressed more doubts in terms of the open-ended questions to the interviewer were as follows: about 54% of all the open-ended questions concerned generic drugs, more than 15% concerned side effects and pharmacovigilance, about 5% of the questions were pertinent to drug control. Other open-ended questions concerned the information leaflet, the economic value of the drug, natural products, vaccines, the pharmacological research.

**Figure 4:**
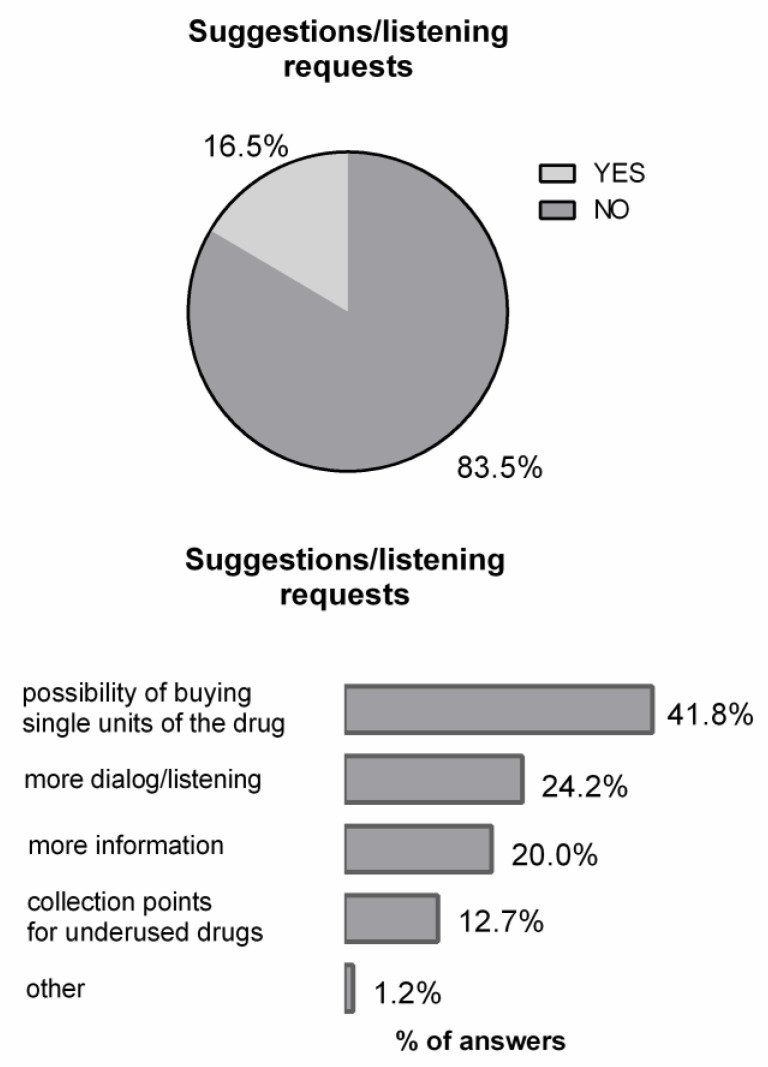
Charts related to spontaneous suggestions and listening requests. Sample size, *n* = 1024.

Figure 4 shows the percentage of the interviewed persons who spontaneously made requests for suggestions/listening and the kinds of the most recurrent spontaneous requests from the respondents (about 1000 interviewees (random sampling over a shorter period of time). The most recurrent spontaneous request of “more dialog/listening”, closely connected with “more information” (their sum was about 44% of all the requests), is consistent with a recent document of the Pharmaceutical Group of the European Union (PGEU) reporting on the importance of the patient-centred approach to healthcare services and the benefits that new technologies can provide [18].

Based on what is reported above, it is clear that the developed tool is characterised by many different factors. As a consequence, an accurate assessment of each of them might be difficult and not fully indicative due to their strict interactions. However, for the purpose of having an answer to the first aspect to test with this pilot study, i.e., the ability of the new tool to provide information about the approach of citizens to drugs meaningful from the pharmacological point of view, the responses of the interviewees were firstly subjected to a preliminary accurate analysis in aggregate by a part of the group of researchers who designed and developed the tool. Then, a board of three members with proven experience in pharmacology from the Institute of Translational Pharmacology who did not participate in the development and implementation of the pilot study was asked to give an opinion on the intelligibility of the selected extensive list of aggregated results. The board expressed a positive opinion, also encouraging the possible dissemination of the same results. Regarding the second aspect we wanted to test with this pilot study, i.e., the possible impact of an awareness plan, an estimation of the potential effectiveness of the plan as a whole was drawn on the basis of answers of the citizens especially included at the end of the interview concerning the approach to drugs they would have from that moment and what they said about the perception of the initiative (Supplementary Materials, Figures S6 and S7). In particular, Supplementary Figure S7 refers to the focus on about 1000 interviewees showing how more than 10% of the respondents who manifested disinterest/lack of knowledge for taking into account the drugs’ side effects, pharmacovigilance system, the information leaflet or the economic value of the drugs expressed their intention to change their own approach as a result of the interview. Moreover, more than 10% of the interviewees who reported a self-approach to treatment interruption and self-use of drugs without healthcare professional consultation expressed their intention to change their approach as a result of the interview. In addition, more than 20% of the respondents with lack of interest/lack of knowledge about generic drugs said they got more information following the interview. An evaluation of how the initiative was perceived showed that while for 83.1% of the interviewees, the knowledge of citizens about the drugs was not sufficient and it would take more actions aimed at improving the citizens’ approach to them, for 97.2% of them, initiatives like COFAIS can be a useful contribution to the public benefit (Supplementary Materials, Figures S8 and S9). It should be further noted that the sample size of the interviews and the fact that they were conducted in various different interview spaces and subareas can make the pilot plan representative of the context.

## 4. Discussion

More information and communication are the aspects emerged from several studies and actions concerning public health [13,14,19]. Intriguingly, such an exigency seems even more pronounced in a digitised era due to possible information distortion by social media [20]. The results reported in this pilot study reveal that implementation plans based on oral interviews and dedicated software platforms could represent a novel integrated strategy to stimulate communication on the use of medicines.

The pilot study also highlighted how difficult it was for citizens to memorise details to identify the drug, for instance, the dosage form and the strength. The researchers expressed the importance for citizens of the details necessary to clearly identify the drug [1,21]. Another element of the pilot study was that the interviews allowed the researchers to stress the importance of speed in reporting potential problems concerning a drug to healthcare professionals, and when not done, of recommending to talk to the doctor or the pharmacist who prescribed or suggested the drug. The indication of drug ineffectiveness was included as it was a commonly reported important adverse effect [7,22]. An indication of lack of effect of the drugs collected by the COFAIS platform should be considered not only as an indication of ineffectiveness of a drug that previously proved to be effective, but also as the indication of the negation of the effectiveness of the drug with respect to the expectation. Another point is that among others self-used drugs, even anxiolytics and antibiotics were indicated by the respondents. Moreover, the frequency of taking drugs without consulting could even be underestimated based on the respondents’ answers. The previous studies on the use of drugs were focused on approaches generally concerning either interventions or informatics products to support people’s health. Our strategy merged the two aspects to develop a tool for an awareness plan on the importance of proper use of pharmaceuticals also capable of stimulating interchanges of information. Moreover, in comparison with the previous studies, our strategy was based on a combination of strengths singularly highlighted in several initiatives [4,11,12,13,14,15,16]. In fact, the face-to-face interview of our pilot study (considered the gold standard method) was performed anonymously, a condition indicated as favourable to reduce hesitation to communicate true views. The interview was also organised to give time to the citizen to reflect and for doubts to arise and tailored to the context. For instance, highlighting the importance of reporting doubts and emerging problems to general practitioners, specialist doctors or pharmacists that prescribed or suggested the drugs, a major goal concerning communication was also achieved during the interview by itself. Finally, the software application COFAIS was designed and developed in strict accordance with the interviews in order to support the data analysis and highlight insights, thus reflecting the complexity of the context. These characteristics make the strategy employed clearly original.

The data collected during the pilot study not only confirmed efficiency of the utilised tool, but also provided new information on the problems and needs inherent to the approach of citizens to drugs, also promoting communication among interested actors. As a consequence, a potential educational impact on possible future patient-centred pharmacovigilance actions could also be hypothesised.

## 5. Conclusions

The results obtained in the pilot study reported here show that the utilised integrated tool consisting of such designed oral interviews and a relational database-backed web application is extremely useful for accomplishing the final double goal of promoting communication concerning the use of medicines by citizens by also providing evidence of critical issues and needs. Thus, an awareness plan organised in such a manner and based on the COFAIS software and oral interviews seems adequate to emphasise problems in the use of pharmaceuticals in the area involved and encourage communication among the different interested actors through the direct involvement of citizens. Based on the results reported here, planning and implementation of such conceived awareness-raising activities capable of supporting further investigation of similar subjects have been considered.

## Data Availability

Not applicable.

## Supplementary Materials

The following materials are available online at www.mdpi.com/article/10.3390/healthcare9111409/s1, Figure S1: Answers to the question “Did you take other drugs or natural products in the same period when the problems occurred?”, Figure S2: Aggregated data on the potential drug problems most frequently indicated during the interviews, Figure S3: Answers to the question “Did you previously take a branded drug?”, Figure S4: Answers to the questions (a) “Do you take pharmaceuticals without consulting your doctor or pharmacist?”, (b) “Have you ever interrupted treatment without consulting your doctor?” and (c) “Do you check if you already have it at home before buying a drug?”, Figure S5: Answers to the question “Do you use generic drugs?”, Figure S6: Answers to the question “Do you think that pointing out problems concerning drugs could contribute to their improvement?”, Figure S7: Answer charts concerning what the respondents said at the beginning of the interview compared to what they said later as a consequence of the interview. Focus on about 1000 interviewees, about 500—for the answer chart concerning information leaflets, Figure S8: Answers to the question “Do you think that the knowledge of citizens about drugs is sufficient?”, Figure S9: Answers to the question “Do you think that an initiative like COFAIS can be a useful contribution to public benefit?”.
