# Supplementary figures and images for: A New Tool for an Awareness Plan Concerning Critical Issues, Needs and Attitudes of Citizens on the Use of Medicines
Source: Healthcare (Basel). 2021 Oct 20;9(11):1409. doi: 10.3390/healthcare9111409 (PMC8619083; doi:10.3390/healthcare9111409)

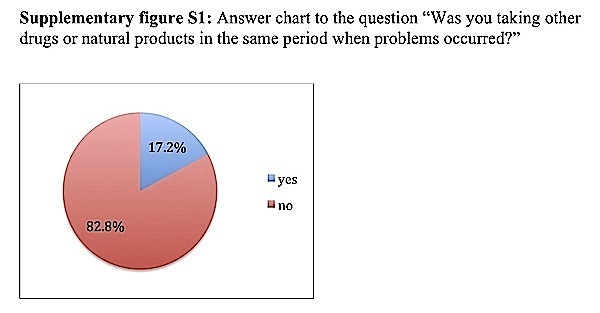

Supplement: Supplementary file 1 [file healthcare-09-01409-s001.zip › healthcare-1335397-supplementary/Figure S1.jpg]

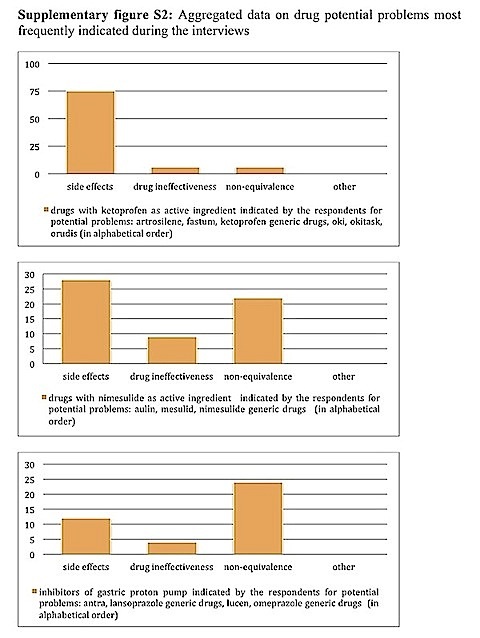

Supplement: Supplementary file 1 [file healthcare-09-01409-s001.zip › healthcare-1335397-supplementary/Figure S2.jpg]

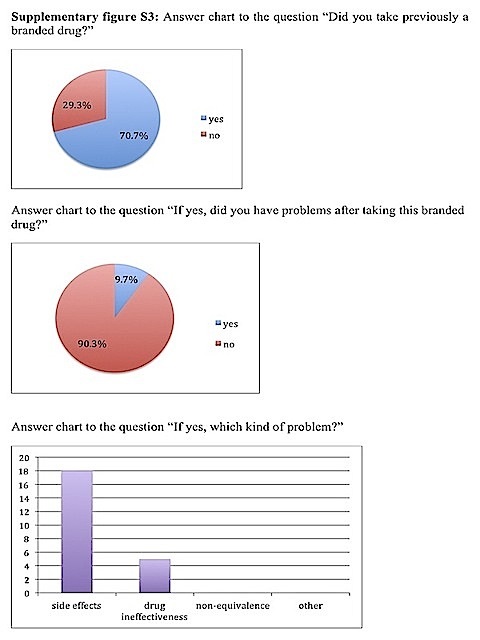

Supplement: Supplementary file 1 [file healthcare-09-01409-s001.zip › healthcare-1335397-supplementary/Figure S3.jpg]

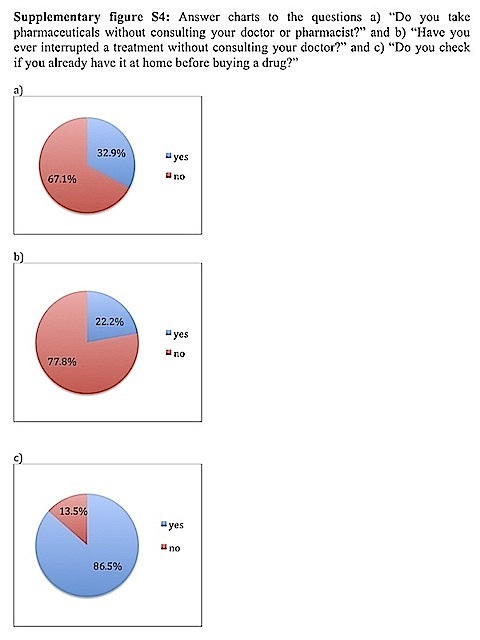

Supplement: Supplementary file 1 [file healthcare-09-01409-s001.zip › healthcare-1335397-supplementary/Figure S4.jpg]

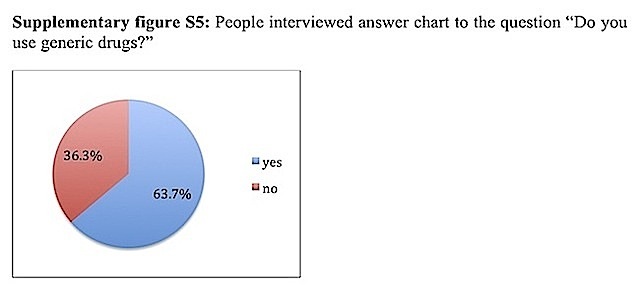

Supplement: Supplementary file 1 [file healthcare-09-01409-s001.zip › healthcare-1335397-supplementary/Figure S5.jpg]

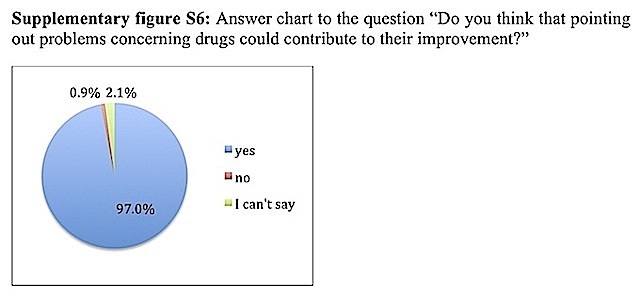

Supplement: Supplementary file 1 [file healthcare-09-01409-s001.zip › healthcare-1335397-supplementary/Figure S6.jpg]

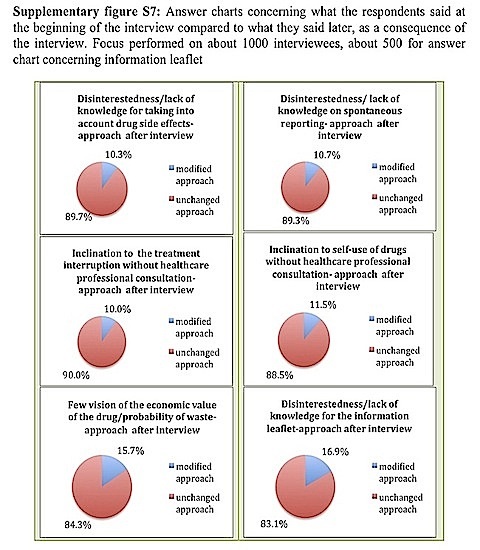

Supplement: Supplementary file 1 [file healthcare-09-01409-s001.zip › healthcare-1335397-supplementary/Figure S7.jpg]

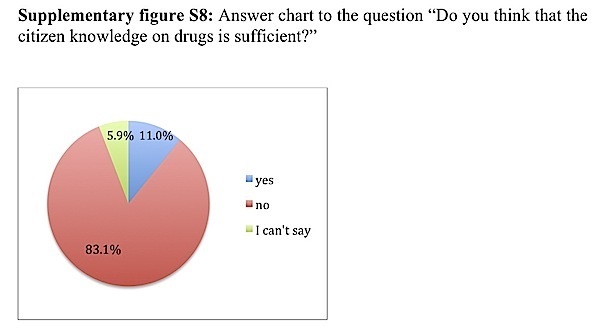

Supplement: Supplementary file 1 [file healthcare-09-01409-s001.zip › healthcare-1335397-supplementary/Figure S8.jpg]

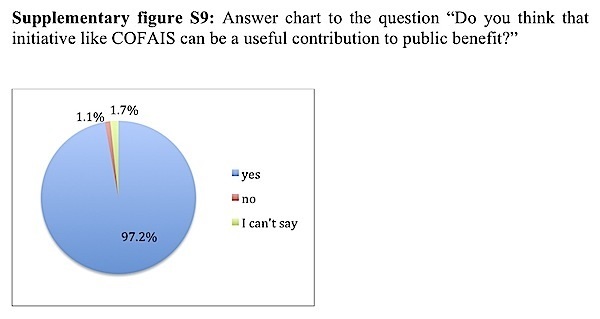

Supplement: Supplementary file 1 [file healthcare-09-01409-s001.zip › healthcare-1335397-supplementary/Figure S9.jpg]
